# Cashew nut (*Anacardium occidentale* L.) and cashew nut oil reduce cardiovascular risk factors in adults on weight-loss treatment: a randomized controlled three-arm trial (Brazilian Nuts Study)

**DOI:** 10.3389/fnut.2024.1407028

**Published:** 2024-06-26

**Authors:** Talitha Silva Meneguelli, Ana Claudia Pelissari Kravchychyn, Aline Lage Wendling, Ana Paula Dionísio, Josefina Bressan, Hercia Stampini Duarte Martino, Elad Tako, Helen Hermana Miranda Hermsdorff

**Affiliations:** ^1^Laboratory of Clinical Analysis and Genomics, Department of Nutrition and Health, Universidade Federal de Viçosa (UFV), Viçosa, Brazil; ^2^Laboratory of Energy Metabolism and Body Composition (LAMECC), Department of Nutrition and Health, Universidade Federal de Viçosa, Viçosa, Brazil; ^3^Brazilian Agricultural Research Corporation (Embrapa) Agroindústria Tropical—CNPAT, Brasília, Brazil; ^4^Laboratory of Experimental Nutrition, Department of Nutrition and Health, Universidade Federal de Viçosa (UFV), Viçosa, Brazil; ^5^Trace Minerals and Nutrition Lab, Department of Food Science, Cornell University, Ithaca, NY, United States

**Keywords:** clinical trial, cashew nut, *Anacardium occidentale* L., obesity, body fat, cardiometabolic markers, liver markers

## Abstract

**Introduction:**

Cashew nut contains bioactive compounds that modulate satiety and food intake, but its effects on body fat during energy restriction remains unknown. This study aimed to assess the effects of cashew nut and cashew nut oil on body fat (primary outcome) as well as adiposity, cardiometabolic and liver function markers (secondary outcomes).

**Materials and methods:**

An eight-week (8-wk) randomized controlled-feeding study involved 68 adults with overweight/obesity (40 women, BMI: 33 ± 4 kg/m^2^). Participants were randomly assigned to one of the energy-restricted (−500 kcal/d) groups: control (CT, free-nuts), cashew nut (CN, 30 g/d), or cashew nut oil (OL, 30 mL/d). Body weight, body composition, and blood collection were assessed at the baseline and endpoint of the study.

**Results:**

After 8-wk, all groups reduced significantly body fat (CT: −3.1 ± 2.8 kg; CN: −3.3 ± 2.7 kg; OL: −1.8 ± 2.6 kg), body weight (CT: −4.2 ± 3.8 kg; CN: −3.9 ± 3.1 kg; OL: −3.4 ± 2.4 kg), waist (CT: −5.1 ± 4.6 cm; CN: −3.9 ± 3.9 cm; OL: −3.7 ± 5.3 cm) and hip circumferences (CT: −2.9 ± 3.0 cm; CN: −2.7 ± 3.1 cm; OL: −2.9 ± 2.3 cm). CN-group reduced liver enzymes (AST: −3.1 ± 5.3 U/L; ALT: −6.0 ± 9.9 U/L), while the OL-group reduced LDL-c (−11.5 ± 21.8 mg/dL) and atherogenic index (−0.2 ± 0.5). Both intervention groups decreased neck circumference (CN: −1.0 ± 1.2 cm; OL: −0.5 ± 1.2 cm) and apo B (CN: −6.6 ± 10.7 mg/dL; OL: −7.0 ± 15.3 mg/dL).

**Conclusion:**

After an 8-wk energy-restricted intervention, all groups reduced body fat (kg), weight, and some others adiposity indicators, with no different effect of cashew nut or cashew nut oil. However, participants in the intervention groups experienced additional reductions in atherogenic marker, liver function biomarkers, and cardiovascular risk factors (neck circumference and apo B levels), with these effects observed across the OL group, CN group, and both intervention groups, respectively.

**Clinical trial registration:**https://ensaiosclinicos.gov.br/rg/RBR-8xzkyp2, identifier 8xzkyp2.

## Graphical Abstract

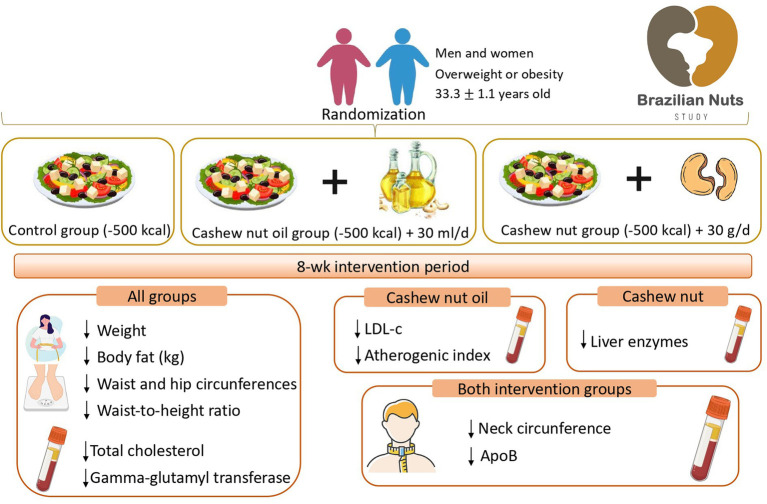

## 1 Introduction

Obesity is a multifactorial and complex disease characterized by excessive adiposity. It is linked to an elevated risk of developing other chronic conditions, including type 2 diabetes mellitus (T2DM), hypertension, dyslipidemia, cardiovascular diseases (CVDs), and some types of cancer (1). This condition represents a burgeoning global pandemic, with estimates indicating that by 2030, over 1 billion people worldwide will be affected by obesity (body mass index (BMI) ≥ 30 kg/m^2^). This projection translates to approximately one in five women and one in seven men (2). In 2019, obesity played a contributing role in around 5 million deaths attributed to cardiovascular diseases, diabetes, cancers, neurological disorders, chronic respiratory diseases, and digestive disorders (3).

One potential strategy for mitigating obesity involves dietary approaches aimed at achieving an optimal energy balance and energy-restriction as treatment (4, 5). Besides, a growing body of evidence from epidemiological studies and clinical trials supports the potential benefits of nuts. Not only do they avoid causing weight gain, but they also seem to contribute to improved body composition and reduced cardiometabolic risk through favorable effects on lipid profiles (6–10).

Among all nuts, cashew nut is one of the most produced and consumed globally, ranking third in both categories (11). In addition to their unsaturated fat content as monounsaturated fatty acids (MUFA) ≈ 62% and polyunsaturated fatty acids (PUFA) ≈ 18%, cashew nut are whole foods that offer supplementary non-lipid nutrients, including proteins (≈21%), dietary fiber (≈4%), and phenolic compounds (≈60 mg GAE/100 g) (12, 13). Furthermore, a derivative of cashew nut, the oil extracted from these nuts, shows promise for promoting health. Cashew nut oil contains high content of monounsaturated fatty acids (MUFA) (≈61%) (14), PUFA ≈ 19%, vitamin E (2225.93 μg/100 g), besides tocopherols and phytosterols (15). Hence, the oil can be positioned as a new product with enhanced value, attributable to its distinctive sensory characteristics, substantial nutritional advantages, and chemical stability (14). However, the combined effects of an energy-restricted diet and the dietary intake of cashew nuts has been not reported, nor has the effect of cashew nut oil on human health.

Thus, we hypothesized that cashew nut and cashew nut oil could contribute to body fat loss and further improvements in body composition, cardiometabolic and liver function markers. The objective of this study was to assess the effects of both cashew nut and cashew nut oil over an 8 week energy restriction on body fat (primary outcome) and other adiposity indicators, cardiometabolic, and liver function markers among adults with overweight/obesity. The findings of this study can contribute to science by providing new insights into the effects of nuts on weight loss and cardiometabolic risk. This study stands out as the first to assess the effect of cashew nuts associated with an energy-restricted diet, as well as the effects of cashew nut oil on overall health. This allows us to compare the effects of cashew nuts in terms of their lipid fraction against other components, such as the whole nut. This unique approach provides a more complete and detailed understanding of the potential health benefits associated with incorporating this nut into the diet. Furthermore, the study analyzed the proximate composition, minerals, fatty acid profile, and phenolic compounds of both cashew nut and cashew nut oil.

## 2 Materials and methods

### 2.1 Cashew nut and cashew nut oil

Cashew nut (*Anacardium occidentale* L.) and cashew nut oil were produced in Brazil, coming from donation of the Brazilian Agricultural Research Corporation (Embrapa), Agroindústria Tropical, Fortaleza (Brazil).

All procedures described next were carried out at the Embrapa. Oil samples were extracted by centrifugation. For sample preparation, the cashew nut was roasted at 110°C for 15 min; cashew nut was ground in a food processor; adding water to the cashew nut (4,1 cashew nut, water w/w) and the mixture was homogenized in a processor at 90°C for 10 min. This mixture was centrifuged for 1 h at 4,500 rpm at room temperature. After centrifugation, the oil was heated in an oven at 105°C for 1 h (14). The raw material was obtained from the same crop, and its microbiological quality was analyzed and assured via reports by the supplier company until they were delivered to the Laboratory of Energy Metabolism and Body Composition of the Universidade Federal de Viçosa (LAMECC/UFV).

For the intervention, cashew nuts were portioned into laminated and vacuum-sealed packages (30 g), while cashew nut oil was fractionated and stored in 250 mL amber glass bottles. Both foods were stored in a freezer at −20°C until distribution to participants to avoid nutrient oxidation, sensory changes, and microbiological contamination. All material for consumption was handled following hygienic-sanitary standards, including the use of clean lab coats, caps, masks, and disposable gloves.

Regarding nutrients and bioactive compounds of cashew nut, moisture, ash, protein, lipids, carbohydrates, dietary fibers, amino acids, and *in vitro* digestibility were evaluated. The moisture, ash, and protein contents were performed according to the methodology indicated by the AOAC (16), the last one was obtained by combustion in the Nitrogen/Protein Analyzer equipment. Carbohydrate content was calculated by the difference of 100 and the sum of the values obtained for moisture, ash, proteins, and lipids. The energy value per 100 g of each product was calculated using the Atwater system: Caloric value = (g of protein × 4) + (g of lipids × 9) + (g of carbohydrates × 4). Total dietary fiber (soluble and insoluble fiber) was determined by the gravimetric non-enzymatic method, using the commercial kit (Total dietary fiber assay kit, Sigma^®^, San Luis, Missouri, EUA) (16). The amino acid contents (aspartic acid, glutamic acid, serine, glycine, histidine, taurine, arginine, threonine, alanine, proline, tyrosine, valine, methionine, cystine, isoleucine, leucine, phenylalanine, lysine, hydroxyproline, tryptophan and the sum of total amino acids) were performed based on the MA-009 R0 method (17, 18), and tryptophan by the MA-010 R.1 method (19). *In vitro* digestibility was analyzed by the previously reported method (20).

Both cashew nut and cashew nut oil underwent analysis for minerals, vitamin E and its derivatives, total phenolics, and antioxidant capacity. Mineral analyzes (phosphorus, potassium, calcium, magnesium, selenium, sodium, copper, iron, zinc, and manganese) were performed according to the methodology of the Food and Drug Administration (FDA) (21). The preparation and analysis of the vitamin E isomers (α-, β-, γ-, δ-tocopherols and tocotrienols) were extracted according to Pinheiro-Sant’Ana et al. (2011), and performed in five replicates by High-Performance Liquid Chromatography (HPLC). During analysis, the samples were protected from sunlight and artificial light using amber glassware, aluminum foil, and blackout curtains, and protected from oxygen by using lids and environments with nitrogen gas in glass bottles. The total phenolic compound content was obtained from reading of absorbance in a spectrophotometer (Thermo Scientific, Evolution 606, United States) at 765 nm. Analytical curve of gallic acid (0.005–0.10 mg/mL) was used to quantify the compounds. The results were expressed in mg of gallic acid equivalents/g of cashew nut (mg GAE/g). The antioxidant activity was determined by the sequestering capacity of free radical DPPH (2,2-diphenyl-1-picryl-hydrazil) as described before (22).

Additionally, we also performed analysis of fatty acids, acidity level, and peroxide index in cashew nut oil. Lipids were obtained using the high-pressure, high-temperature extraction system in Ankom XT-15 equipment according to the American Oil Chemists’ Society (23). The fatty acid profile was determined using the procedure described by Hartman and Lago (1973). The determinations of acidity and peroxides were performed according to AOCS (2003).

### 2.2 Trial design

This is an 8-wk randomized controlled three-arm dietary intervention, in which subjects were assigned to receive control (CT), cashew nut (CN) or cashew nut oil (OL) plus an energy-restricted diet. This study was conducted at the Department of Nutrition and Health of the Universidade Federal de Viçosa (UFV), Brazil, between January 2022 and July 2022, according to the guidelines laid down in the Declaration of Helsinki. All procedures involving human subjects were approved by the Ethics Committee in Research with Human Experimentation of the Universidade Federal de Viçosa (No. 4.543.541/CEPH). Written informed consent was obtained from all subjects/patients. The study is registered at the Brazilian Registry of Clinical Trials (ReBEC) with ID number RBR-8xzkyp2.

**Figure 1:**
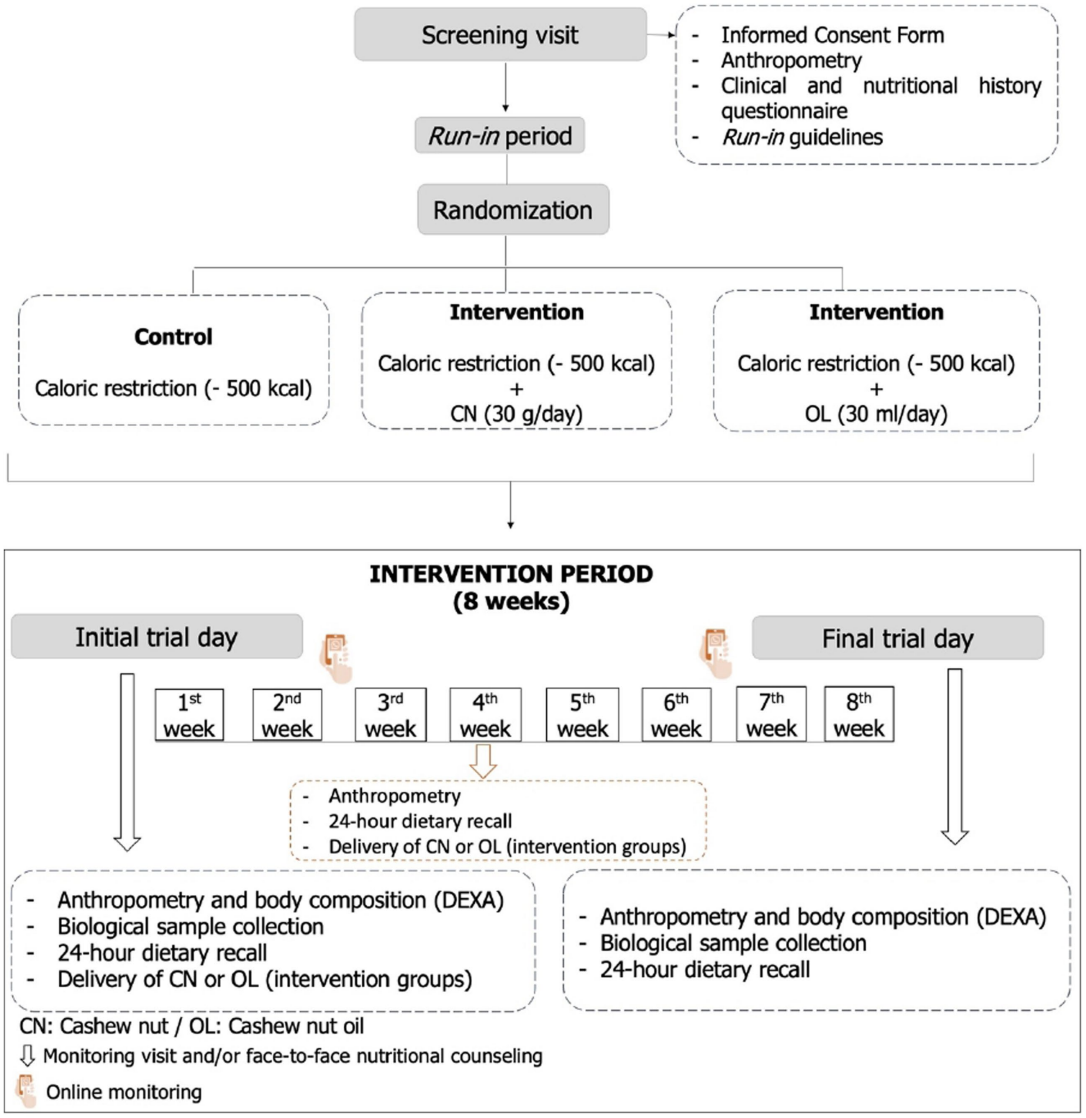
Study design flowchart. Source: own elaboration.

During the intervention, participants attended on three occasions at the LAMECC/UFV: initial and final days for blood sample collection, anthropometry, body composition evaluation and fill out questionnaires about physical activity practice and food record, and in the fourth week (30 days) for a face-to-face monitoring visit and anthropometric measurements. Between face-to-face visits, participants received online monitoring (Figure 1).

Compliance was evaluated during the monitoring visit to determine whether participants were adhering to the intervention. We tracked the consumption of cashew nut and cashew nut oil from the initial day of the intervention until their return. If participants in the intervention group still possessed cashew nuts or oil from the beginning of the study upon returning, they were required to surrender them for assessment, verifying whether they had indeed consumed the prescribed quantity. Additionally, during the application of the return questionnaire, we evaluated whether participants had commenced any new medication or developed any illnesses. The participants who started taking any drug or developed any disease listed in the non-inclusion criteria was excluded from the study. Furthermore, compliance was gauged at the study’s conclusion by monitoring weight gain. Since the intervention aimed to body fat loss by energy restriction, participants were expected to experience weight loss. Consequently, individuals who exhibited weight gain were excluded from the study due to non-compliance.

### 2.3 Study participants

Study participants were recruited in Viçosa, Minas Gerais, Brazil, via radio announcements, social media, and the UFV network platform. An online form assessed individuals’ eligibility, with eligible candidates undergoing a face-to-face selection questionnaire to confirm eligibility. This questionnaire covered clinical, dietary, sociodemographic, and anthropometric data, along with body composition, blood pressure, and recent biochemical test results. Participants received a booklet containing guidelines about the study and were instructed to report any changes in medication or health status.

The inclusion criteria for participants in the study consisted of men or women (20–55 y); with overweight (27–29.9 kg/m^2^), waist circumference (WC) ≥80 cm for women and ≥90 cm for men and with body fat percentage >30% for women and >20% for men associated with at least another component of metabolic syndrome (MS): triglycerides (TG) ≥150 mg/dL; blood pressure ≥130/85 mmHg or fasting blood glucose ≥100 mg/dL or who uses medication to control these markers; or men or women with obesity (BMI ≥ 30 kg/m^2^), WC ≥80 cm for women and ≥90 cm for men, and body fat percentage >30% for women and >20% for men with or without metabolic complications.

The non-inclusion criteria included pregnant, lactating, or menopausal women; athletes; vegans; or have a diagnosis of insulin-dependent diabetes; diagnosis of HIV, digestive, hepatic, renal, cardiovascular, thyroid, cancer, inflammatory diseases and eating disorders; history of drug and/or alcohol abuse; have an aversion or allergy to nuts; present infection in the last month; habitually consume nuts above 30 g/day; use drugs such as anti-inflammatories, corticosteroids, and antibiotics, capable of biochemical alterations; chewing difficult; weight instability (5% of usual weight) in the last 3 months; alcohol consumption >21 units (≈168 g) per week; and intake of vitamin, mineral, and omega 3 supplements.

### 2.4 Run-in

One week before intervention, the participants participated in a run-in period. During run-in the subjects were instructed to consume their habitual diets without nuts, dried fruits like berries (cranberry, blueberry, goji berry and raisins), açaí, cocoa, cinnamon, olive oil and alcoholic beverages, and to maintain their usual activities. Following the run-in period, individuals whose body weight fluctuated beyond ±1 kg or who consumed prohibited foods or beverages were categorized as “poor responders” and excluded from the study.

### 2.5 Intervention

All participants received an energy-restricted (−500 kcal/d) diet. In addition, the cashew nut group received 30 g/d of vacuum-sealed cashew nut to be consumed daily, and the cashew nut oil group received 250 mL amber glass bottles of oil along with a measuring cup to standardize the amount to 30 mL/d of cashew nut oil. All dietary advice was individualized and provided by dietitians. At the beginning, five energy-restricted diet options for all groups were designed and divided into five meals: breakfast, morning snack, lunch, afternoon snack, and dinner. All menus were calculated in an Excel spreadsheet using the Brazilian Institute of Geography and Statistics (IBGE) table. Energy requirements were calculated according to the Mifflin’s formula (24). For everyone, 500 kcal were reduced from the total calculated energy requirement, considering the level of physical activity of each participant. For the interventional groups, a daily cashew nut (30 g/d) or cashew nut oil (30 mL/d) was added to the individual meal plans, and the percentage of energy from total fat was around 27% for cashew nut group, 32% for the cashew nut oil group, while the control group had around 21% (Table 1). This amount of cashew nut was based on previous studies that have used similar amounts, the PREvención con DIeta MEDiterránea (PREDIMED), which demonstrated beneficial effects in the improvement of blood pressure, lipid profile, lipoprotein particles, inflammation, oxidative stress, and carotid atherosclerosis (25, 26). For the cashew nut oil group, 30 mL/d was calculated to reach similar amounts of lipid between the two intervention groups. Moreover, most guidelines recommend a dietary intake ranging from 10 to 25% for monounsaturated fats (MUFA) and from 6 to 11% for polyunsaturated fats (PUFA) (27). As shown in Table 1 of our paper, the prescribed amounts for cashew nut group were 17.38 ± 2.71% for MUFA and 6.41 ± 1.31% for PUFA, and 14.52 ± 2.32% for MUFA and 6.01 ± 1.3% for PUFA for cashew nut oil group, including 30 g/day of cashew nuts and 30 mL/day of cashew nut oil, respectively. These values are in close alignment with the recommended doses, and people can easily consume on a daily basis. There was no statistical difference in energy calculated between the groups (*p* = 0.959).

**Table 1:** Macronutrients, dietary fiber, and energy distribution among dietary intervention groups.

Nutrients	Control	Cashew nut	Cashew nut oil	*p*-value
Total fat (%)	21.19 ± 1.84 ^c^	27.04 ± 2.49 ^b^	31.83 ± 3.87 ^a^	<0.001
Saturated Fat (%)	7.87 ± 1.97 ^b^	10.96 ± 1.51 ^a^	7.78 ± 1.47 ^b^	<0.001
Monounsaturated Fat (%)	5.97 ± 1.58 ^c^	17.38 ± 2.71 ^a^	14.52 ± 2.32 ^b^	<0.001
Polyunsaturated Fat (%)	3.57 ± 0.86 ^b^	6.41 ± 1.31 ^a^	6.01 ± 1.33 ^a^	<0.001
Carbohydrates (%)	55.26 ± 3.51 ^a^	48.06 ± 4.17 ^b^	47.17 ± 5.55 ^b^	<0.001
Proteins (%)	23.55 ± 2.74 ^a^	24.89 ± 3.14 ^a^	20.99 ± 3.15 ^b^	<0.001
Dietary fiber (g)	25.90 ± 7.69	22.62 ± 7.02	22.64 ± 8.49	0.065
Energy (kcal)	1600.83 ± 318.29	1607.40 ± 307.82	1618.05 ± 317.15	0.959

Superscript alphabets ^(a–c)^ not indicated by the same letter means statistical difference between groups (*p* < 0.005) according to one-way ANOVA or Kruskal–Wallis followed by post hoc tests. Letter a represents the highest value, while letter c is the lowest.

Participants were instructed to incorporate cashew nut as a mid-morning snack, while those assigned to the cashew nut oil group were provided with recipes for incorporating the oil into shakes and salad dressings. Members of the cashew nut and cashew nut oil groups (intervention) were explicitly directed not to use the cashew nut or their oil for cooking, roasting, or frying purposes. Additionally, they were advised against consuming olive oil, avocado, or any other nuts aside from the allocated quantity of cashew nut, as well as any other foods with high unsaturated fat content. Control group participants were similarly instructed to refrain from consuming any type of nuts, olive oil, avocado, or other foods high in unsaturated fat.

### 2.6 Outcomes

The primary outcome of the trial was a change in body fat. Secondary outcomes were changes in the values of body weight, BMI, waist, hip and neck circumferences, waist-to-hip ratio (WHR), waist-to-height ratio (WHtR), cardiometabolic (TG, total cholesterol, LDL-c, HDL-c, VLDL-c, ApoA1, ApoB, cortisol, total cholesterol:HDL-c, LDL-c:HDL-c) and liver function markers (AST, ALT, GGT, alkaline phosphatase) after 8 weeks of follow-up.

Body composition was assessed by dual-energy X-ray absorptiometry (Lunar Prodigy Advance DXA System, GE Lunar) and provided fat mass (FM), fat-free mass (FFM), lean mass (LM), and total mass were obtained from the total body and regions, such as trunk, android, and gynoid. The android area is between the ribs and the pelvis, while the gynoid region includes the hips and upper thighs and overlaps the leg and truncal regions. The body composition in percentages was calculated in relation to total body measurements. Body weight was assessed by a bioelectrical impedance analysis device (Inbody 230, Biospace Corp.). Height (meters), waist, hip, and neck circumferences (centimeters) were measured according to standard protocols. BMI was calculated as weight divided by squared height (kg/m^2^) according to World Health Organization (WHO) (28). WHR was calculated as waist divided by hip circumference, and WHtR was calculated as waist divided by height.

Fasting (10–12 h) venous whole blood samples were collected by a registered nurse at baseline and the end of the study (8-wk) into vacuum tubes containing EDTA as an anticoagulant. Then, blood samples were centrifugated (3,500 r.p.m., 10 min, 4°C), separated in aliquots and stored until analysis. The biochemical determinations were performed by the Hemolab clinical analysis laboratory (Viçosa-MG, Brazil). Trained nursing technicians, specifically employed for this project, conducted the blood collection, obtaining samples ranging from 20 to 30 mL via vacuum. Samples were collected for evaluation of cardiometabolic risk as TG (≥150 mg/dL), total cholesterol (≥240 mg/dL), LDL-c (≥160 mg/dL), HDL-c (<40 or <50 mg/dL for men and women, respectively), and VLDL-c (≥30 mg/dL). Also, apolipoprotein-A-1 (APO-A-1), apolipoprotein-B (APO-B), liver markers such as AST transaminase, gamma GT, ALT transaminase, and alkaline phosphatase were compared as mean and standard deviation between groups. Besides, the atherogenic indices, total cholesterol:HDL-c and LDL-c:HDL-c proposed by Castelli (1988) were calculated.

### 2.7 Dietary assessments

At baseline and the end of the study, we applied a 24 h recall (24HR) to monitor food consumption during the intervention. The reported intake was analyzed using the 24HR-ERICA software, adapted for the Brazilian population, and the IBGE table (29, 30).

### 2.8 Sample size and study power

The sample size and study power were determined using the G*Power 3.1 program. For this calculation, a total of 57 volunteers were determined, based on an average estimated effect size derived from clinical studies (0.30), considering statistical analyses for three groups, two intervention points (baseline and endpoint), an alpha value set at 0.05, and a power of 0.80. By adding 20% as a result of losses during follow-up, the total sample size was determined to be 68 participants (Supplementary Figure S1).

For the power of the study, the effect size of 0.28 was calculated from the Eta squared (0.074) based on the values of body fat from our database, an *α* of 0.05 was used, three groups, two intervention points (baseline and endpoint), and the total sample size of 68 individuals, whom we have information on body fat data. The calculation revealed a study power of 0.94 (Supplementary Figure S2).

### 2.9 Randomization

To initiate the intervention, after the run-in period, researchers performed the randomization using MinimPy 0.3 program (31). This was achieved through the stratified minimization method, accounting for sex, age, and BMI, with three levels per factor. This approach ensured a well-balanced distribution of potential factors that could interfere with the outcome variables.

### 2.10 Statistical analysis

Statistical analysis was conducted using SPSS version 22.0 (SPSS, Inc.), and figures displaying statistical analysis were produced using Microsoft Excel. A *p*-value <0.05 was considered statistically significant. The Shapiro–Wilk test was performed to check the normality of variables. Data are expressed as mean values and standard deviation. Among groups, variable changes were compared by one-way ANOVA followed by Tukey’s *post hoc* test or using the non-parametric Kruskal–Wallis test followed by Dunn’s *post hoc* test. To compare differences between baseline and post-intervention within the groups, pairwise tests were performed (paired *t*-test or Wilcoxon). McNemar’s test was employed to analyze paired nominal data.

## 3 Results

### 3.1 Cashew nut and cashew nut oil

Regarding minerals, the content of calcium (CN: 0.37 g/kg vs. OL: 0.01 g/kg) and iron (CN: 64.00 mg/kg vs. OL: 6.10 mg/kg) was higher in cashew nut compared to the oil. Other minerals were not detected in the oil. The oil demonstrated elevated amounts of vitamin E (OL: 2225.93 μg/100 g vs. CN: 1334.02 μg/100 g) and γ tocopherol (OL: 2055.12 μg/100 g vs. CN: 1334.02 μg/100 g) compared to cashew nut. Additionally, β tocopherol, γ tocotrienol, and δ tocotrienol, which were not present in cashew nut, were found in the oil. Conversely, cashew nut exhibited higher levels of total phenolics (CN: 60.45 vs. OL: 2.25 mg GAE (gallic acid equivalent)/100 g) and antioxidant capacity (CN: 15.99 vs. OL: 9.18 uM TE/g sample) in comparison to their oil (Supplementary Tables S1, S2).

### 3.2 Participants and compliance

**Figure 2:**
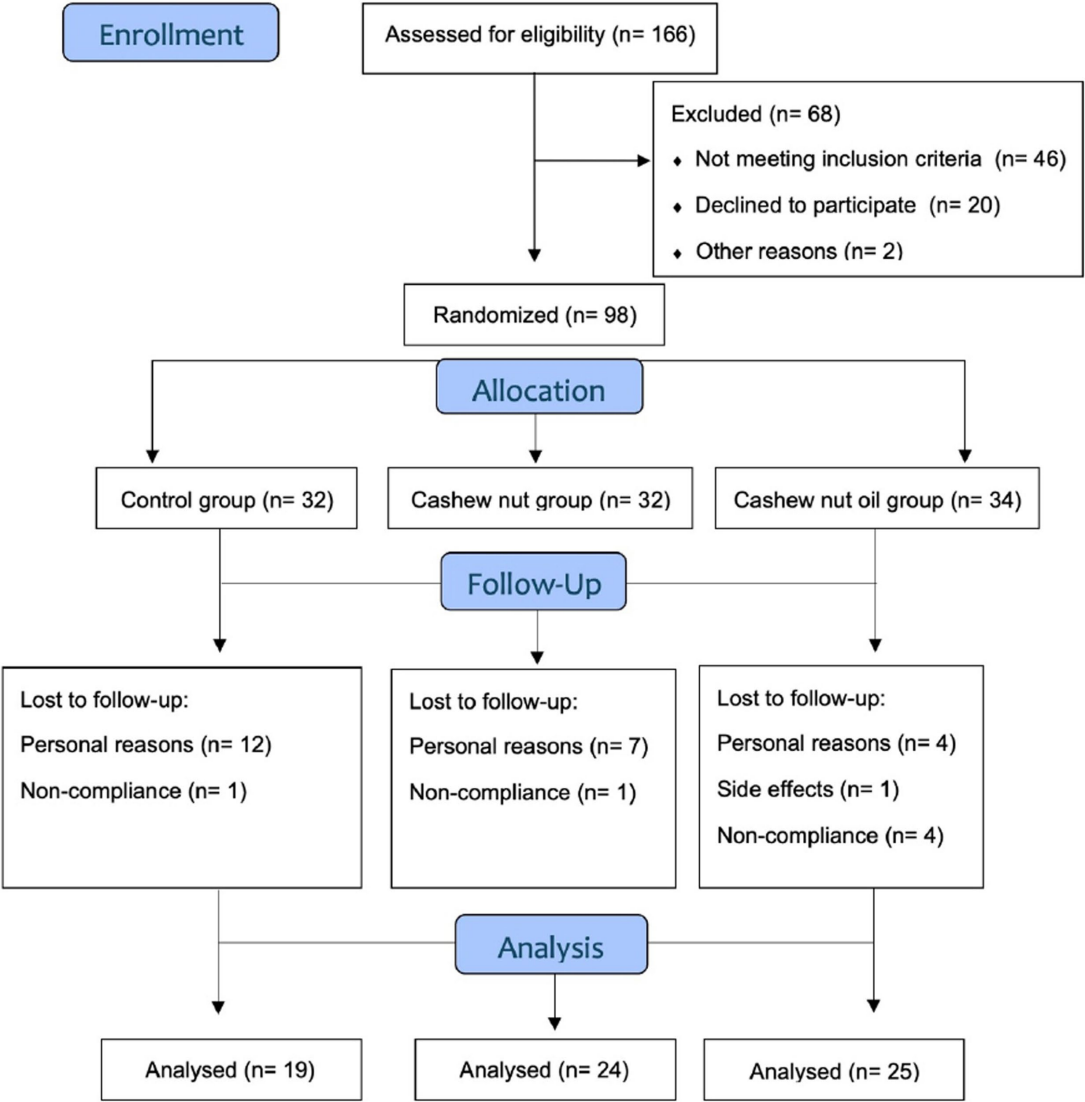
Consort statement of participants flow diagram.

Among the participants initially assessed for study eligibility (*n* = 166), 98 were included and randomly assigned to the following groups: CT (*n* = 32), CN (*n* = 32) and OL (*n* = 34). Of these, 74 participants completed the 8-wk intervention, allocated as follows: CT (*n* = 20), CN (*n* = 25) and OL (*n* = 29). Of these 74 participants, six participants were subsequently excluded due to non-compliance with the prescribed diet as they gained weight. Since all participants were on a low-energy diet, weight loss was expected. Consequently, those who concluded the study with weight gain were excluded due to non-compliance, resulting in the following numbers for analysis in each group: CT (*n* = 19), CN (*n* = 24) and OL (*n* = 25) (Figure 2).

The study population predominantly comprised females (*n* = 40), individuals with a completed college education/incomplete postgraduate (*n* = 27), white (*n* = 33), self-reported single marital status (*n* = 39), and a family income between 2 and 3 minimum wages (*n* = 23). Regarding lifestyle habits, the majority did not smoke (*n* = 65) and did not engage in regular physical activity (*n* = 43) (Table 2). Concerning cardiometabolic risk at baseline, 19 (79.2%), 15 (78.9%), and 23 (92%) individuals had obesity, while 13 (54.2%), 11 (57.9%), and 13 (52%) had dyslipidemia in the CN, CT, and OL groups, respectively (data not shown).

**Table 2:** Sociodemographic and behavioral characteristics of the total participants according to the control and intervention groups (cashew nut and cashew nut oil).

**Variables**	**Total**	**Control**	**Cashew nut**	**Cashew nut oil**	***p*-value**
	**(*n*=68)**	**(*n*= 19)**	**(*n*= 24)**	**(*n*= 25)**	
Age (years)	33.31 ± 8.75	34.68 ± 9.65	33.79 ±8.39	31.80 ± 8.50	0.53
Sex:					
*Male*	28 (41.2)	10 (35.7)	9 (32.1)	9 (32.1)	0.49
*Female*	40 (58.8)	9 (22.5)	15 (37.5)	16 (40)	
Smoking					
*Yes*	3 (4.4)	1 (33.3)	1 (33.3)	1 (33.3)	0.98
*No*	65 (95.6)	18 (27.7)	23 (35.4)	24 (36.9)	
Physically active					
*Yes*	25 (36.8)	5 (20)	10 (40)	10 (40)	0.53
*No*	43 (63.2)	14 (32.6)	14 (32.6)	15 (34.9)	
Schooling					
*Complete primary education / Incomplete high school*	2 (2.9)	0 (0)	0 (0)	2 (100)	0.09
*Complete high school / Incomplete college education*	23 (33.8)	7 (30.4)	6 (26.1)	10 (43.5)	
*Complete college education / Incomplete postgraduate*	27 (39.7)	10 (37)	8 (29.6)	9 (33.3)	
*Complete postgraduate*	16 (23.5)	2 (12.5)	10 (62.5)	4 (25)	
Family income					
*1 minimum wage*	3 (4.4)	2 (66.7)	0 (0)	1 (33.3)	0.55
*1 to 2 minimum wages*	16 (23.5)	4 (25)	3 (18.8)	9 (56.3)	
*2 to 3 minimum wages*	23 (33.8)	7 (30.4)	9 (39.1)	7 (30.4)	
*3 to 5 minimum wages*	15 (22.1)	4 (26.7)	6 (40)	5 (33.3)	
*5 to 10 minimum wages*	7 (10.3)	1 (14.3)	3 (42.9)	3 (42.9)	
*> 10 minimum wages*	3 (4.4)	1 (33.3)	2 (66.7)	0 (0)	
Race					
*White*	33 (48.5)	8 (24.2)	12 (36.4)	13 (39.4)	0.92
*Black*	15 (22.1)	4 (26.7)	6 (40)	5 (33.3)	
*Pardo*	20 (29.4)	7 (35)	6 (30)	7 (35)	
Marital status					
*Single*	39 (57.4)	9 (23.1)	11 (28.2)	19 (48.7)	**0.02**
*Married/stable partnership*	27 (39.7)	10 (37)	13 (48.1)	4 (14.8)	
*Divorced*	2 (2.9)	0 (0)	0 (0)	2 (100)	

For age values, one-way ANOVA followed by post hoc tests were used and the results are represented by mean ± SD (standard deviation). For all other variables, the chi-square test was used, and the results are presented in absolute and relative frequency values as shown in the table as n (%).

Following the 8-wk intervention, all participants in the study demonstrated a reduction in energy intake (−205 kcal; *p* = 0.026), indicating adherence to energy-restriction (data not shown). However, this reduction was not as substantial as expected (−500 kcal). When examining the groups individually, the control group exhibited a reduction in the intake of saturated fat (SFA), while the cashew nut group experienced a decrease in polyunsaturated fat (PUFA) and α-linolenic acid (Table 3).

**Table 3:** Food consumption according to 8-wk energy-restricted intervention groups.

Daily Nutrient Intake	Control (*n* = 17)			Cashew nut (*n* = 20)			Cashew nut oil (*n* = 23)		
	Baseline	Δ	*p*-value	Baseline	Δ	*p*-value	Baseline	Δ	*p*-value
Energy intake (kcal)	1667.7 ± 560.3	−301.3 ± 692.8	0.114	1663.3 ± 592.6	−32.5 ± 685.4	0.838	1670.9 ± 671.8	−284.6 ± 660.6	0.051
Protein (% EI)	20.1 ± 6.9	−1.2 ± 5.9	0.455	20.60 ± 6.3	0.7 ± 6.4	0.640	17.7 ± 6.7	1.8 ± 8.2	0.312
Carbohydrate (% EI)	44.2 ± 10.5	6.4 ± 13.1	0.078	46.1 ± 10.3	−0.3 ± 13.5	0.920	47.2 ± 8.6	−2.4 ± 12.5	0.378
Lipids (% EI)	35.8 ± 8.8	−4.1 ± 11.4	0.186	34.1 ± 8.2	−0.2 ± 13.2	0.957	35.7 ± 8.8	1.4 ± 11.7	0.572
SFA (g)	13.4 ± 4.8	−2.5 ± 4.2	0.037	12.4 ± 3.00	0.1 ± 6.1	0.959	11.9 ± 3.5	−0.3 ± 5.6	0.819
MUFA (g)	12.3 ± 3.7	−0.9 ± 5.8	0.540	11.5 ± 3.9	1.6 ± 5.8	0.249	12.7 ± 4.4	1.3 ± 6.2	0.332
PUFA (g)	5.6 ± 1.3	−0.1 ± 2.9	0.892	6.7 ± 3.1	−2.0 ± 3.4	0.019	6.2 ± 3.00	−0.2 ± 3.8	0.799
LA (C18:2n6) (g)	9.2 ± 4.2	−1.9 ± 6.3	0.240	10.3 ± 6.7	−2.7 ± 6.7	0.100	10.1 ± 6.5	−1.7 ± 8.8	0.374
ALA (C18:3n3) (g)	0.6 ± 0.9	−0.1 ± 1.1	0.696	1.2 ± 1.7	−0.9 ± 1.8	0.037	0.9 ± 1.1	−0.3 ± 1.2	0.324
Cholesterol (mg)	387.8 ± 276.5	−137.3 ± 262.7	0.062	341.2 ± 293.8	−31.3 ± 296.4	0.651	328.6 ± 226.5	9.7 ± 304.5	0.880
Fiber (g)	17.6 ± 11.1	1.8 ± 10.9	0.522	17.3 ± 7.2	0.9 ± 8.4	0.644	15.6 ± 7.7	0.4 ± 6.7	0.786

∆ = endpoint − baseline assessment. Values are mean ± SD (standard deviation). *p*-value = intra-group comparison (paired *t*-test). According to one-way ANOVA or Kruskal–Wallis followed by post hoc tests there was no statistical difference between groups. EI, energy intake; SFA, saturated fatty acid; MUFA, monounsaturated fatty acid; PUFA, polyunsaturated fatty acid. LA (C18:2n6): linoleic acid. ALA (C18:3n3): α-linolenic acid.

### 3.3 Body fat and adiposity indicators

After 8-wk of intervention, all groups presented significant reduction in body fat (kg), with consequent weight-loss (CT: −4.4%; CN: −4.1%; OL: −3.5%). The participants also had significant reduction in other adiposity indicators as: weight-loss, WC (cm), hip circumference (HC) (cm), and WHtR. Concerning WHR and body fat (%), significant losses were observed only in the control and cashew nut groups. Both intervention groups (cashew nut and oil) exhibited a significant reduction in neck circumference. No differences were found between groups, except for android fat in the endpoint between CT and OL groups (CT: 9.5 ± 1.3%; OL: 8.1 ± 1.5%) (Table 4). Additionally, after the intervention, there was a decrease in the number of individuals with obesity among those who consumed cashew nut (19 (27.94%) vs. 13 (19.12%); *p* = 0.032) (Supplementary Figure S3).

**Table 4:** Change in body fat and other adiposity indicators according to 8-wk energy-restricted intervention groups.

Outcomes		Baseline	Endpoint	Δ	*p*-value (intraindividual)
		(*n* = 68)	(*n* = 68)		
Body fat (kg)	CT	38.5 ± 7.7	35.4 ± 8.1	−3.1 ± 2.8	<0.001
	CN	41.5 ± 8.9	38.2 ± 9.1	−3.3 ± 2.7	<0.001
	OL	41.1 ± 7.9	39.3 ± 8.9	−1.8 ± 2.6	0.002
*p*-value (interindividual)		0.357	0.345	0.106	
Body fat (%)	CT	40.1 ± 7.8	38.5 ± 8.2	−1.6 ± 1.6	<0.001
	CN	43.6 ± 7.6	41.8 ± 8.4	−1.9 ± 1.9	<0.001
	OL	43.8 ± 7.6	43.2 ± 8.4	−0.7 ± 2.0	0.106
*p*-value (interindividual)		0.307	0.206	0.085	
Android fat (%)	CT	9.7 ± 1.3	9.5 ± 1.3 ^a^	−0.2 ± 0.6	0.233
	CN	9.4 ± 3.4	8.7 ± 1.7 ^ab^	−0.8 ± 2.6	0.169
	OL	8.5 ± 1.4	8.1 ± 1.5 ^b^	−0.4 ± 1.1	0.072
*p*-value (interindividual)		0.122	0.022	0.579	
Gynoid fat (%)	CT	17.2 ± 1.8	17.3 ± 1.8	0.1 ± 0.7	0.463
	CN	18.9 ± 3.6	18.2 ± 1.5	−0.7 ± 3.5	0.360
	OL	17.8 ± 2.3	18.2 ± 1.9	0.3 ± 1.2	0.182
*p*-value (interindividual)		0.353	0.214	0.372	
Muscle mass (kg)	CT	54.3 ± 13.0	53.8 ± 12.5	−0.5 ± 1.8	0.943
	CN	50.9 ± 11.3	50.4 ± 11.4	−0.4 ± 1.7	0.241
	OL	50.2 ± 12.1	48.9 ± 11.6	−1.2 ± 2.0	0.005
*p*-value (interindividual)		0.753	0.472	0.582	
Body weight (kg)	CT	95.4 ± 17.2	91.2 ± 15.9	−4.2 ± 3.8	<0.001
	CN	96.1 ± 14.8	92.2 ± 13.8	−3.9 ± 3.1	<0.001
	OL	95.6 ± 14.3	92.2 ± 13.8	−3.4 ± 2.4	<0.001
*p*-value (interindividual)		0.982	0.944	0.852	
BMI (kg/m^2^)	CT	33.7 ± 3.7	32.3 ± 3.7	−1.4 ± 1.2	<0.001
	CN	34.1 ± 4.9	32.7 ± 4.8	−1.4 ± 1.0	<0.001
	OL	33.9 ± 3.6	32.8 ± 3.8	−1.2 ± 0.8	<0.001
*p*-value (interindividual)		0.978	0.804	0.833	
WC (cm)	CT	109.5 ± 9.5	104.4 ± 7.7	−5.1 ± 4.6	<0.001
	CN	109.3 ± 11.7	105.4 ± 11.7	−3.9 ± 3.9	<0.001
	OL	107.7 ± 12.1	103.9 ± 10.5	−3.7 ± 5.3	0.002
*p*-value (interindividual)		0.838	0.884	0.612	
HC (cm)	CT	113.8 ± 5.7	110.8 ± 7.0	−2.9 ± 3.0	<0.001
	CN	116.6 ± 7.3	113.9 ± 7.4	−2.7 ± 3.1	<0.001
	OL	116.8 ± 6.6	113.8 ± 6.2	−2.9 ± 2.3	<0.001
*p*-value (interindividual)		0.283	0.271	0.902	
WHR	CT	0.9 ± 0.0	0.9 ± 0.1	−0.01 ± 0.0	
	CN	0.9 ± 0.1	0.9 ± 0.1	−0.01 ± 0.0	0.010
	OL	0.9 ± 0.1	0.9 ± 0.1	−0.01 ± 0.0	0.264
*p*-value (interindividual)		0.186	0.323	0.552	
WHtR	CT	0.7 ± 0.1	0.6 ± 0.1	−0.03 ± 0.0	0.003
	CN	0.7 ± 0.1	0.6 ± 0.1	−0.02 ± 0.0	<0.001
	OL	0.6 ± 0.1	0.6 ± 0.1	−0.02 ± 0.0	0.003
*p*-value (interindividual)		0.837	0.887	0.591	
Neck circumference (cm)	CT	40.6 ± 3.5	39.6 ± 3.5	−0.9 ± 2.5	0.104
	CN	39.9 ± 4.5	38.9 ± 3.8	−1.0 ± 1.2	<0.001
	OL	38.9 ± 4.4	38.4 ± 4.1	−0.5 ± 1.2	0.038
*p*-value (interindividual)		0.438	0.555	0.238	

BMI, body mass index; HC, hip circumference; WHR, waist-to-hip ratio; WHtR, waist-to-height ratio; CT, control; CN, cashew nut; OL, cashew nut oil. Paired *t* test or Wilcoxon test (*p* < 0.05 within-group); Superscript alphabets (^a–b^) not indicated by the same letter in the same column means that there was a statistical difference between groups (*p* < 0.05) according to one-way ANOVA or Kruskal–Wallis followed by post hoc tests.

### 3.4 Cardiometabolic and liver function markers

After 8-wk intervention, all groups reduced total cholesterol and GGT. In the intervention groups, both the cashew and oil groups had reductions in apo B, while those consuming only the oil experienced reductions in LDL-c and the atherogenic index (total cholesterol/HDL-c). The control and cashew nut groups observed reductions in TG and VLDL-c. In terms of liver enzymes, the cashew nut group demonstrated reductions in AST and ALT. No differences were found between groups (Table 5).

**Table 5:** Change in cardiometabolic and liver function markers according to 8-wk energy-restricted intervention groups.

Biomarkers		Baseline (*n* = 68)	Endpoint (*n* = 68)	Δ	*p*-value (intraindividual)
Cardiometabolic markers					
Triglycerides (mg/dL)	CT	176.4 ± 98.4	124.1 ± 79.2	−52.3 ± 46.8	<0.001
	CN	127.9 ± 58.8	96.9 ± 45.6	−30.9 ± 46.2	0.003
	OL	142.2 ± 89.2	120.9 ± 74.9	−21.3 ± 48.9	0.055
*p*-value (interindividual)		0.119	0.284	0.052	
Total cholesterol (mg/dL)	CT	194.3 ± 30.9	181.6 ± 37.2	−12.7 ± 23.9	0.046
	CN	186.2 ± 31.6	172.4 ± 32.4	−13.8 ± 24.4	0.011
	OL	207.2 ± 46.7	190.8 ± 39.8	−16.4 ± 30.4	0.007
*p*-value (interindividual)		0.133	0.266	0.996	
LDL-c (mg/dL)	CT	103.1 ± 30.3	101.6 ± 31.6	−1.5 ± 25.4	0.616
	CN	105.9 ± 30.1	100.1 ± 30.9	−5.8 ± 18.5	0.141
	OL	121.4 ± 34.7	109.9 ± 30.3	−11.5 ± 21.8	0.016
*p*-value (interindividual)		0.119	0.357	0.316	
HDL-c (mg/dL)	CT	56.1 ± 11.1	55.1 ± 12.2	−1.0 ± 6.3	0.636
	CN	54.3 ± 9.8	52.9 ± 9.1	−1.5 ± 6.7	0.216
	OL	57.4 ± 11.5	56.8 ± 13.5	−0.6 ± 5.4	0.464
*p*-value (interindividual)		0.735	0.694	0.895	
VLDL-c (mg/dL)	CT	35.3 ± 19.7	24.8 ± 15.8	−10.5 ± 9.7	0.002
	CN	25.8 ± 11.4	19.4 ± 9.1	−4.3 ± 9.8	0.002
	OL	28.4 ± 17.8	24.2 ± 14.9	−3.7 ± 10.2	0.055
*p*-value (interindividual)		0.119	0.284	0.050	
ApoA1 (mg/dL)	CT	127.9 ± 18.8	123.8 ± 17.9	−4.1 ± 10.1	0.111
	CN	120.8 ± 12.1	117.2 ± 14.4	−3.6 ± 15.7	0.123
	OL	127.0 ± 19.6	124.6 ± 22.7	−2.4 ± 12.1	0.368
*p*-value (interindividual)		0.583	0.543	0.912	
ApoB (mg/dL)	CT	87.8 ± 15.1	84.4 ± 22.2	−3.4 ± 13.1	0.103
	CN	84.7 ± 17.4	78.1 ± 18.3	−6.6 ± 10.7	0.003
	OL	93.7 ± 25.1	86.6 ± 21.3	−7.0 ± 15.3	0.020
*p*-value (interindividual)		0.291	0.322	0.980	
Cortisol (mcg/dL)	CT	13.0 ± 3.3	13.7 ± 4.7	0.7 ± 4.7	0.546
	CN	14.9 ± 6.892	14.9 ± 5.570	0.02 ± 6.4	0.753
	OL	14.4 ± 5.9	15.0 ± 5.6	0.67 ± 5.8	0.502
*p*-value (interindividual)		0.899	0.664	0.909	
Atherogenic indices					
Total cholesterol:HDL-c	CT	3.6 ± 0.8	3.4 ± 0.8	−0.2 ± 0.4	0.091
	CN	3.5 ± 0.7	3.4 ± 0.9	−0.1 ± 0.4	0.076
	OL	3.7 ± 0.9	3.5 ± 0.7	−0.2 ± 0.5	0.026
*p*-value (interindividual)		0.833	0.742	0.741	
LDL-c:HDL-c	CT	1.9 ± 0.6	1.9 ± 0.6	0.02 ± 0.5	0.968
	CN	1.9 ± 0.6	1.9 ± 0.7	−0.03 ± 0.4	0.394
	OL	2.2 ± 0.7	2.0 ± 0.6	−0.2 ± 0.4	0.072
*p*-value (interindividual)		0.297	0.757	0.310	
Liver markers					
AST (U/L)	CT	26.7 ± 12.4	22.5 ± 7.5	−4.3 ± 11.7	0.098
	CN	24.7 ± 6.5	21.5 ± 5.5	−3.1 ± 5.3	0.007
	OL	25.5 ± 8.9	26.5 ± 14.9	1.0 ± 11.7	0.945
*p*-value (interindividual)		0.884	0.825	0.400	
ALT (U/L)	CT	25.7 ± 9.7	24.1 ± 12.6	−1.6 ± 11.7	0.314
	CN	26.8 ± 14.2	20.8 ± 8.7	−6.0 ± 9.9	<0.001
	OL	25.9 ± 12.4	25.0 ± 14.4	−0.9 ± 7.8	0.375
*p*-value (interindividual)		0.924	0.671	0.240	
GGT (U/L)	CT	39.8 ± 14.8	32.8 ± 13.8	−7.0 ± 10.7	0.005
	CN	42.9 ± 30.9	31.4 ± 16.4	−11.5 ± 20.3	<0.001
	OL	39.0 ± 19.1	31.4 ± 13.9	−7.1 ± 10.8	0.005
*p*-value (interindividual)		0.746	0.690	0.902	
Alkaline phosphatase (U/L)	CT	79.2 ± 21.0	79.1 ± 17.9	−0.1 ± 7.1	0.679
	CN	81.4 ± 23.5	82.9 ± 25.1	1.5 ± 12.1	0.661
	OL	76.9 ± 21.5	79.1 ± 19.5	2.2 ± 11.1	0.277
*p*-value (interindividual)		0.781	0.561	0.784	

LDL-c, low-density lipoprotein-cholesterol; HDL-c, high-density lipoprotein cholesterol; VLDL-c, very-low-density lipoprotein; AST, aspartate aminotransferase; ALT, alanine transaminase; GGT, gamma-glutamyl transferase; ApoA1, apolipoprotein A1; ApoB, apolipoprotein B; CT, control; CN, cashew nut; OL, cashew nut oil. Paired *t*-test or Wilcoxon test (*p* < 0.05 within-group); Superscript alphabets (^a–b^) not indicated by the same letter in the same column means that there was a statistical difference between groups (*p* < 0.05) according to one-way ANOVA or Kruskal–Wallis followed by post hoc tests.

## 4 Discussion

In this clinical trial, all groups demonstrated a reduction in body fat (kg) and other total adiposity (body weight and BMI) and central adiposity indicators (WC, HC and WHtR), as well as in total cholesterol and GGT. Additionally, both intervention groups (CN and OL) experienced a decrease in neck circumference and apo B, but not control group. Cashew nut group reduced liver enzymes (AST and ALT), while cashew nut oil group reduced LDL-c and atherogenic index. Furthermore, there was a reduction in the number of individuals with obesity in the group consuming cashew nut. However, no differences were found between groups.

We expected that the presence of cashew nut or cashew nut oil would exert a greater reduction in body fat, and other adiposity indicators, as well as cardiometabolic markers compared to control group. Thus, the results of this study were not consistent with our hypothesis. Several factors are crucial for contributing to weight loss, with chewing time playing a pivotal role in satiety due to its impact on neural and endocrine mechanisms. The effort involved in oral consumption and the duration spent chewing whole nuts have been linked to significant effects on satiety, the presence of fat in meals, and the stimulation of postprandial hormones such as insulin, ghrelin, CCK, PYY, and GLP-1 (32), which has previously been discussed by our research group (33). As oil has a liquid form, its digestion and absorption are quicker, abbreviating the duration of satiety. A study showed that satiety increased after chewing whole walnuts compared to walnut butter, although gut peptide concentrations remained unchanged (34). Nonetheless, although we standardized the timing of cashew nut consumption among all participants, we did not regulate the duration of chewing, which made a detailed discussion on this aspect impossible. Thus, to gain a comprehensive understanding of the effects of cashew nut consumption on satiety in future studies, it is important to incorporate a protocol that specifies chewing duration. Previous findings from our research group indicated a decrease in ghrelin hormone levels among those who consumed a mix of nuts (30 g of cashew nuts +15 g of Brazil nuts) compared to the control group (35). Confounding factors may also have affected the results, notably the inadequate adherence to the prescribed diet. Despite a prescribed caloric reduction of 500 kcal, the observed reduction was only 205 kcal, as previously demonstrated. This indicates that, overall, participants did not adhere to the diet as intended, potentially impacting the results irrespective of the interventions.

Our previous research supports the findings of this study concerning adiposity indicators and other cardiometabolic markers. We demonstrated that both the control group and the group consuming a mix of nuts (30 g of cashew nuts +15 g of Brazil nuts), alongside an energy-restricted diet for 8 weeks, experienced reductions in total and central adiposity indicators and other cardiometabolic markers, with no statistically significant differences between the groups. However, exceptions were observed in body fat (%) and VCAM-1 levels, where a statistically significant difference emerged, indicating a reduction in the group that consumed the mix of nuts compared to an increase in the control group (36).

Nuts appear to not promote an increase in adiposity markers, while the reduction of these markers is still controversy, depending on the type of nut and intervention design (37). A meta-analysis has shown that almonds were able to reduce body weight and fat mass, but not waist circumference (38). On the other hand, walnuts and cashews did not significantly modify adiposity indicators (39, 40). However, it is important to highlight that there are relatively few studies evaluating the health effects of cashew nuts compared to other nuts such as almonds, walnuts, pistachio, and peanuts (37, 40–42). Despite this, a meta-analysis presented an interesting result when comparing the duration of nut intake interventions (<12 weeks vs. ≥12 weeks), showing sustained significance in reductions of body weight, BMI, and WC in individuals with overweight and obesity when the intervention duration was ≥12 weeks, in contrast to durations of <12 weeks (37). This result leads us to consider that perhaps if the duration of our study were ≥12 weeks, we could find differences between the intervention groups compared to the control group, especially considering our target population (individuals with overweight and obesity), since the result demonstrated by this meta-analysis was for this specific group.

While our study did not uncover any statistically significant differences among the three groups, both cashew nuts and cashew nut oil demonstrated a potential in improving cardiovascular risk. This was evidenced by a statistically significant reduction in neck circumference and apo B levels observed in both intervention groups, which was not observed in the control group. The neck circumference is an indicator of subcutaneous fat distribution (43). A larger neck circumference is suggestive of higher levels of body fat, including visceral adipose tissue, which poses a risk for cardiovascular disease (44). The group that consumed cashew nuts experienced an average reduction of −1.0 ± 1.2 cm in neck circumference, while the group that consumed cashew nut oil experienced an average reduction of −0.5 ± 1.2 cm. Although these reductions may appear minor, they hold significance due to the neck perimeter’s sensitivity as a health indicator. This is supported by the fact that 1 cm increase in neck circumference can lead to a rise in the risk of obesity by 1.21 to 1.73%, of systolic blood pressure (SBP) by 1.06 to 1.10%, and of diastolic blood pressure (DBP) by 1.08 to 1.06% (45). Additionally, it can elevate the risk of diabetes by 1.04 to 1.10%, hypertriglyceridemia by 1.04 to 1.10%, and metabolic syndrome by 1.08 to 1.28% (45). Thus, even modest reductions in neck circumference can exert a significant influence on decreasing cardiovascular risk. Other studies found that daily nut consumption led to decreases in LDL-c by 4.2 mg/dL and apo B levels by 4.1 mg/dL (4 and 6% reduction in coronary events, respectively) (46). Both pistachios and almonds reduced apo B levels (47, 48), while pistachios also lowered LDL-c levels (47). A meta-analysis involving twenty-five randomized controlled trials (RCTs) along with four newer RCTs and a controlled parallel trial showed that reducing SFA intake while increasing MUFA intake leads to a reduction in plasma apoB and LDL-C. However, the findings are less consistent concerning to plasma TAG, HDL-C, apoA1, and the apoB:apoA1 ratio (49). As evidenced, a decrease of 4.1 mg/dL in cholesterol levels leads to a 6% reduction in coronary events. Within the scope of our study, we observed a reduction of 6.6 mg/dL in the group that consumed cashew nuts and 7.0 mg/dL in the group that ingested cashew nut oil. This finding has significant clinical implications, suggesting a potential impact on the prevention of future coronary events.

Another important outcome of this study was the significant reduction in LDL concentrations and the atherogenic index (total cholesterol:HDL) observed in the OL-group. These markers are closely associated with cardiovascular risk (50). LDL-c, recognized as an atherogenic lipoprotein, plays a pivotal role in the development and progression of atherosclerosis. The TC:HDL-C ratio is considered a more valuable marker for determining Coronary Heart Disease (CHD) risk, being more sensitive and specific than total cholesterol as a risk predictor (51, 52).

Cashew nut, with their high content of MUFA and PUFA and low levels of SFAs, have previously been associated with LDL-lowering effects (53). Our study found a PUFA/SFA ratio of 1.04 ± 0.01 (Table 1). This ratio indicates the potential of a food to contribute to fat accumulation in body tissues when consumed. The Department of Health and Social Security advises avoiding edible oils with a PUFA/SFA ratio below 0.45 (15). Therefore, the cashew nut oil used in our clinical trial is considered beneficial for the human diet and could help reduce cardiometabolic markers.

**Figure 3:**
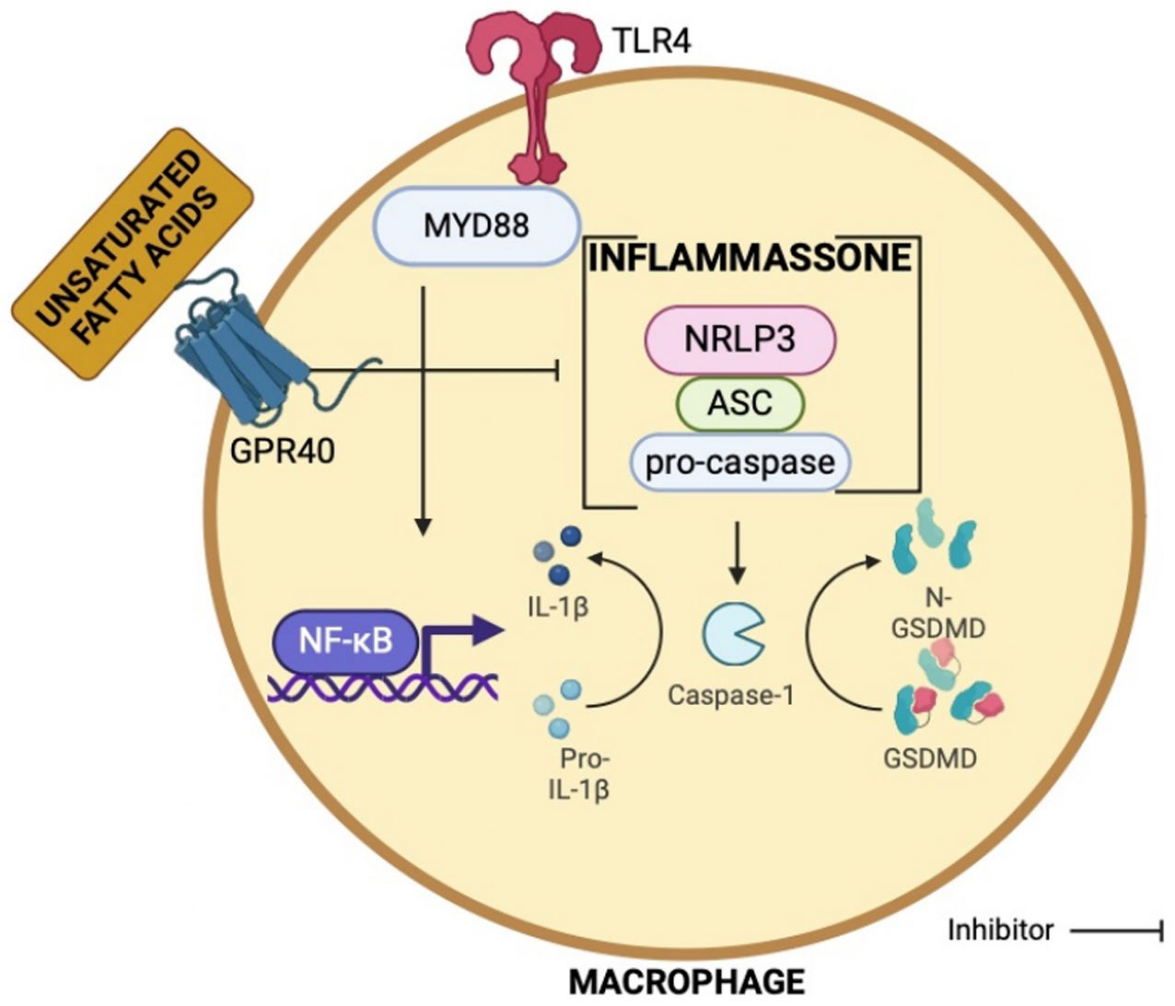
Biomechanical pathway showing the possible effects of cashew nut oil towards improving atherogenic function.

Moreover, oleic acid, the main type of MUFA present in cashew nut, is associated with better cardiovascular health, and may have contributed to the reduction of these markers. Oleic acid exhibits several protective mechanisms in vascular cells (54). Firstly, it increases the levels of uncoupling proteins-2 (UCP-2), which are associated with vascular cell protection, preventing atherosclerosis development (55). Also, oleic acid reduces the activation of JNK1/2, crucial for cardiovascular cells, through its anti-inflammatory action. Unexpectedly, oleic acid has been found to possess anti-inflammatory properties by preventing NLRP3 inflammasome activation (56). Furthermore, oleic acid protects against vascular smooth muscle cell (VSMC) proliferation stimulated by TNF-α, Ang II, or palmitate, thereby contributing to the prevention of atherosclerotic plaque growth (57). Thus, a possible biomechanical pathway was proposed showing the possible effects of cashew nut oil towards improving atherogenic function (Figure 3).

Consistent with our findings, the PREDIMED study revealed that the consumption of olive oil, particularly the extra-virgin variety, was associated with reduced risks of cardiovascular disease and mortality in individuals at high cardiovascular risk. The authors attribute these benefits to components present in extra virgin olive oil (EVOO), such as the high content of MUFAs, which are less susceptible to oxidation than other types of fatty acids. Additionally, they point to other minor components with significant biological properties, including phenolic compounds, vitamin E, and other lipid derivative molecules (such as squalene, tocopherols, and triterpene alcohols), particularly abundant in EVOO (58). Cashew nut oil had significant amounts of both MUFA and vitamin E, whereas cashew nuts were rich in phenolic compounds. It is plausible that these components contributed to the reduction of markers associated with cardiovascular risk.

Another noteworthy outcome of the present study was the reductions in liver enzymes observed in the group that consumed cashew nut. Abnormal levels of liver enzymes have been linked to metabolic disorders such as insulin resistance and diabetes (59). Cashew nut contained higher amounts of magnesium, selenium, and phenolic compounds. Maybe the combination of these elements in cashew nut can contribute for these findings, as some studies indicate that supplementation with magnesium, and selenium can be beneficial to the liver (60–63). Studies have indicated that magnesium is inversely related to non-alcoholic fatty liver disease (NAFLD) (8, 9). In particular, evidence shows that magnesium influences AST and ALT enzymes, as magnesium treatment normalized AST in 87% and ALT in 91% of patients, compared to 57 and 63%, respectively, of the group treated with placebo (10). Phenolic compounds appear to act on the liver X receptor (LXR), which is found mainly in the liver (11). One of their main roles is to regulate cholesterol and lipid metabolism, which makes them ideal targets to prevent or improve dyslipidemia (11). Related to this, pecan shells have high antioxidant potential, and these phenolic compounds present in pecan shells may be involved in reduced lipid peroxidation observed in liver tissue (64). The beneficial actions of phytochemicals are acknowledged for their biologically active polyphenols, such as flavonoids and phenolic acids, which exhibit potent antioxidant activities, including the reduction of lipid peroxidation observed in liver tissue (65). However, whether cashew nuts can affect liver function is unknown. The exact mechanism by which cashew nuts influence biomarkers of liver function remains incompletely understood. Further investigations, mainly using animal models, are needed to elucidate the effect of cashew nuts on the biomechanical pathway in the liver.

Cashew nut and cashew nut oil have the potential to improve cardiometabolic markers. However, the energy-restricted diet alone has also demonstrated substantial health benefits, including weight loss, improved body composition, and lowered levels of total cholesterol and triglycerides. Therefore, when aiming to enhance health, incorporating these two foods should be complemented by a comprehensive and well-balanced eating plan, taking into account the overall nutritional quality of the diet and the bioactive compounds present in the foods. Other studies from our laboratory also demonstrated beneficial effects of nuts on health. The consumption of the mix of nuts enhanced the intestinal microbiota correlating with body fat reduction (66). In this way, our research group has demonstrated some beneficial effects of Brazilian nuts (Brazil nut and cashew nut) during energy-restriction treatment. Furthermore, when we evaluated the acute effects of these nuts, we observed a reduction in oxidative stress, as evidenced by a decrease in malondialdehyde levels, which was positively correlated with the concentrations of TG, VLDL, TG/HDL, and blood pressure (67).

The study has some limitations. First, we find a discrepancy between the planned (−500 kcal) and reported calorie restriction (−205 kcal). This variance is a common challenge in human intervention studies, especially those in free-living condition, when individuals maintain their daily life patterns, in contrast to controlled studies conducted in laboratory settings. Additionally, during the follow-up period, some participants discontinued their participation, a common occurrence in dietary intervention studies due to challenges in altering lifestyle habits and adherence difficulties, as we can see in other randomized controlled trials (68–73).

The study’s strengths include its randomized controlled design, ensuring groups with similar characteristics and reducing selection bias, thereby enhancing the study’s representativeness for the target population. This design also significantly improves the study’s external validity. Adherence to the study protocol was diligently monitored through regular online and face-to-face visits conducted every 15 days. The inclusion of both men and women in the study enhances the extrapolation of results to real-life scenarios, increasing the applicability and relevance of the findings.

Our study contributes to the literature since there are few studies evaluating cashew nut compared to other nuts (e.g. almonds, walnuts, pistachio, and peanuts). Also, this was the first study to evaluate cashew nut oil on health, discerning the benefits of cashew nut arising from its lipid fraction or other non-lipid constituents.

## 5 Conclusion

Individuals in all three groups experienced reduced body weight and other indicators of adiposity over an 8-week period, with no differences between the three groups. Thus, our hypothesis regarding the potential benefits of cashew nut and cashew nut oil on body fat loss, improvements in body composition and cardiometabolic risk has not been confirmed. However, cashew nut group reduced liver enzymes, while cashew nut oil group reduced LDL-c and atherogenic index, and both the group consuming cashew nut or cashew nut oil experienced reductions in neck circumference and apo B after intervention. All these reductions were not statistically significant in the control group. Thus, the study’s findings support the incorporation of cashew nut and cashew nut oil, along with an energy-restricted diet, to have a potential to improve atherogenic and liver function biomarkers in individuals with overweight or obesity. To see differences in body fat and other adiposity as well as cardiometabolic markers between the intervention and control groups, it may be necessary to provide guidance to participants on chewing time and extend the study duration to at least 12 weeks. Since this was the first study to evaluate the impact of cashew nut oil on health, further investigations, particularly focusing on the oil, are needed.

## Data availability statement

The raw data supporting the conclusions of this article will be made available by the authors, without undue reservation.

## Ethics statement

The studies involving humans were approved by Ethics Committee in Research with Human Experimentation of the Universidade Federal de Viçosa (No. 4.543.541/CEPH). The studies were conducted in accordance with the local legislation and institutional requirements. The participants provided their written informed consent to participate in this study.

## Author contributions

TM: Formal analysis, Investigation, Methodology, Writing – original draft. AK: Investigation, Methodology, Writing – review & editing. AW: Investigation, Methodology, Writing – review & editing. AD: Resources, Writing – review & editing. JB: Conceptualization, Writing – review & editing. HM: Writing – review & editing. ET: Writing – review & editing. HH: Conceptualization, Funding acquisition, Methodology, Project administration, Supervision, Writing – review & editing.
