# Supplementary material for: Cashew nut (Anacardium occidentale L.) and cashew nut oil reduce cardiovascular risk factors in adults on weight-loss treatment: a randomized controlled three-arm trial (Brazilian Nuts Study)
Source: Front Nutr. 2024 Jun 26;11:1407028. doi: 10.3389/fnut.2024.1407028 (PMC11234893; doi:10.3389/fnut.2024.1407028)
Supplement: Supplementary file 1 [file Table_1.DOCX]

Supplementary Material

# Supplementary Tables

**Supplementary Table 1** Chemical composition, dietary fiber, minerals, amino acids, digestibility, total phenolics and antioxidant capacity of the cashew nut

| **Cashew nut parameters** | **Values** |
| --- | --- |
| *Centesimal composition* | |
| Moisture (%) | 3.18 ± 0.01 |
| Ashes (%) | 2.69 ± 0.02 |
| Lipidis (%) | 41.98 ± 0.22 |
| Protein (%) | 21.50 ± 0.51 |
| Carbohydrates (%) | 30.65 ± 0.47 |
| Energetic value (kcal/100 g) | 586.41 ± 1.11 |
| *Fibers (%)* | |
| Total | 22.36 ± 0.05 |
| Insoluble | 21.33 ± 0.40 |
| Soluble | 1.03 ± 0.45 |
| *Minerals* |  |
| Phosphorus (g/kg) | 5.36 ± 0.43 |
| Potassium (g/kg) | 7.01 ± 0.43 |
| Calcium (g/kg) | 0.37 ± 0.08 |
| Magnesium (g/kg) | 2.79 ± 0.35 |
| Selenium (g/kg) | 1.71 ± 0.16 |
| Sodium (g/kg) | 0.18 ± 0.02 |
| Copper (mg/kg) | 17.33 ± 1.15 |
| Iron (mg/kg) | 64.00 ± 6.00 |
| Zinc (mg/kg) | 54.00 ± 4.00 |
| Manganese (mg/kg) | 17.33 ± 1.15 |
| Vitamin E total (μg/100g) | 1334.02 ± 150.51 |
| γ tocopherol (μg/100g) | 1334.02 ± 150.51 |
| *Amino acids (%)* | |
| Aspartic Acid | 1.99 |
| Glutamic Acid | 4.7 |
| Serine | 1.18 |
| Glycine | 0.96 |
| Histidine | 0.47 |
| Taurine | LQ |
| Arginine | 2.49 |
| Threonine | 0.8 |
| Alanine | 0.88 |
| Proline | 0.79 |
| Tyrosine | 0.72 |
| Valine | 1.18 |
| Methionine | 0.41 |
| Cystine | 0.42 |
| Isoleucine | 0.87 |
| Leucine | 1.57 |
| Phenylalanine | 0.99 |
| Lysine | 1.01 |
| Hydroxyproline | LQ |
| Tryptophan | 0.3 |
| Sum of Total Amino Acids | 21.73 |
| Crude Protein | 23.24 |
| *In vitro digestibility* | 86.72 ± 0.68 |
| *Total phenolics (mg GAE /100 g sample)* | 60.45 ± 0.86 |
| *Antioxidant capacity (DPPH) (uM TE /g sample)* | 15.99 ± 0.45 |

GAE: Gallic Acid Equivalent; TE: Trolox Equivalent. α tocopherol, β tocopherol, α tocotrienol, β tocotrienol, γ tocotrienol, and δ tocotrienol were not detected. Amounts of taurine and hydroxyproline were identified below the quantification limit.

**Supplementary Table 2.** Acid and peroxide index, fatty acids, total phenolics and antioxidant capacity of cashew nut oil

| **Cashew nut oil parameters** | **Values** |
| --- | --- |
| *Minerals* |  |
| Calcium (g/kg) | 0.01 |
| Iron (mg/kg) | 6.1 |
| Vitamin E (μg/100g) | 2225.93 ± 234.20 |
| β tocopherol (μg/100g) | 48.92 ± 11.57 |
| γ tocopherol (μg/100g) | 2055.12 ± 195.41 |
| γ tocotrienol (μg/100g) | 26.24 ± 3.68 |
| δ tocotrienol (μg/100g) | 95.64 ± 23.54 |
| *Fatty acids (%)* | |
| Mystic (C14:0) | 0.02 ± 0.00 |
| Palmitic (C16:0) | 9.22 ± 0.01 |
| Palmitoleic (C16:1) | 0.28 ± 0.00 |
| Margaric (17:0) | 0.12 ± 0.00 |
| Heptadecenoic (17:1) | 0.05 ± 0.00 |
| Stearic (C18:0) | 8.59 ± 0.01 |
| Oleic (C18:1n-9) | 67.85 ± 0.06 |
| Linoleic (C18:2n-6) | 17.90 ± 0.02 |
| Arachidonic (C20:4n-6) | 0.73 ± 0.00 |
| Gondoic (C20:1n-9) | 0.13 ± 0.00 |
| γ-Linolenic (C18:3n-6) | 0.22 ± 0.00 |
| Heneicosanoic (C21:0) | 0.04 ± 0.00 |
| Behenic (C22:0) | 0.08 ± 0.03 |
| Unidentified | 0.35 ± 0.00 |
| SFA | 18.05 ± 0.02 |
| MUFA | 68.31 ± 0.06 |
| PUFA | 18.85 ± 0.02 |
| PUFA/SFA | 1.04 ± 0.01 |
| *Acidity level (mg KOH/g)* | 0.37 ± 0.03 |
| *Peroxide Index (meq/Kg)* | 0.83 ± 0.16 |
| *Total phenolics (mg GAE / 100 g sample)* | 2.25 ± 0.35 |
| *Antioxidant capacity (DPPH) (uM TE /g sample)* | 9.18 ± 0.81 |

SFA: saturated fatty acid; MUFA: monounsaturated fatty acid; PUFA: polyunsaturated fatty acid; GAE: Gallic Acid Equivalent; TE: Trolox Equivalent. α tocopherol, δ tocopherol, α tocotrienol, and β tocotrienol were not detected. Phosphorus, potassium, magnesium, selenium, sodium, copper, zinc, and manganese were identified below the quantification limit.
