# Supplementary material for: Cashew nut (Anacardium occidentale L.) and cashew nut oil reduce cardiovascular risk factors in adults on weight-loss treatment: a randomized controlled three-arm trial (Brazilian Nuts Study)
Source: Front Nutr. 2024 Jun 26;11:1407028. doi: 10.3389/fnut.2024.1407028 (PMC11234893; doi:10.3389/fnut.2024.1407028)
Supplement: Supplementary file 2 [file Image_1.pdf]

Supplementary Material

1 Supplementary Figures

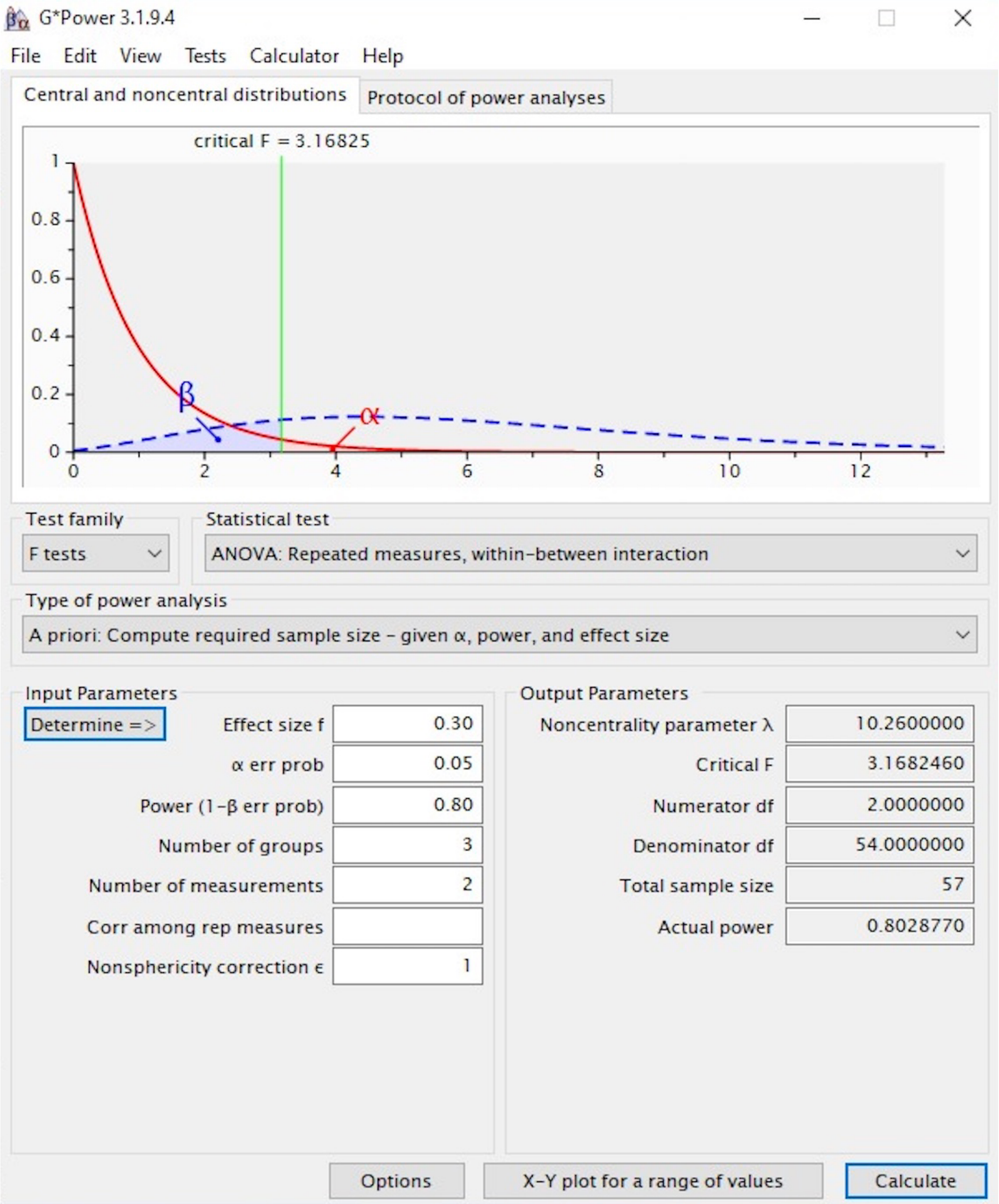

Supplementary Figure 1. Result of sample size calculation according to G*Power 3.1 program.

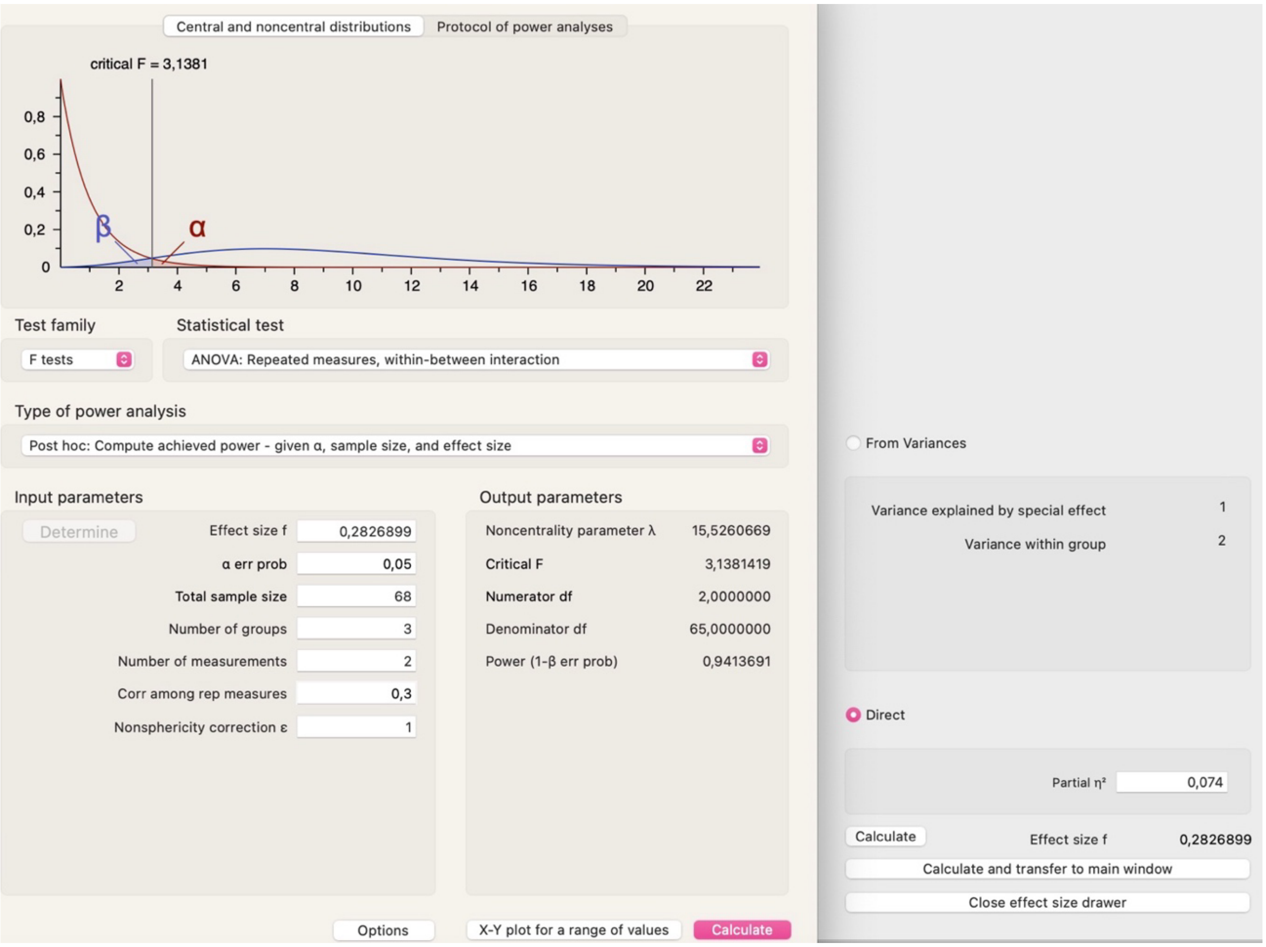

Supplementary figure 2. Study power calculation according to G*Power 3.1 program.

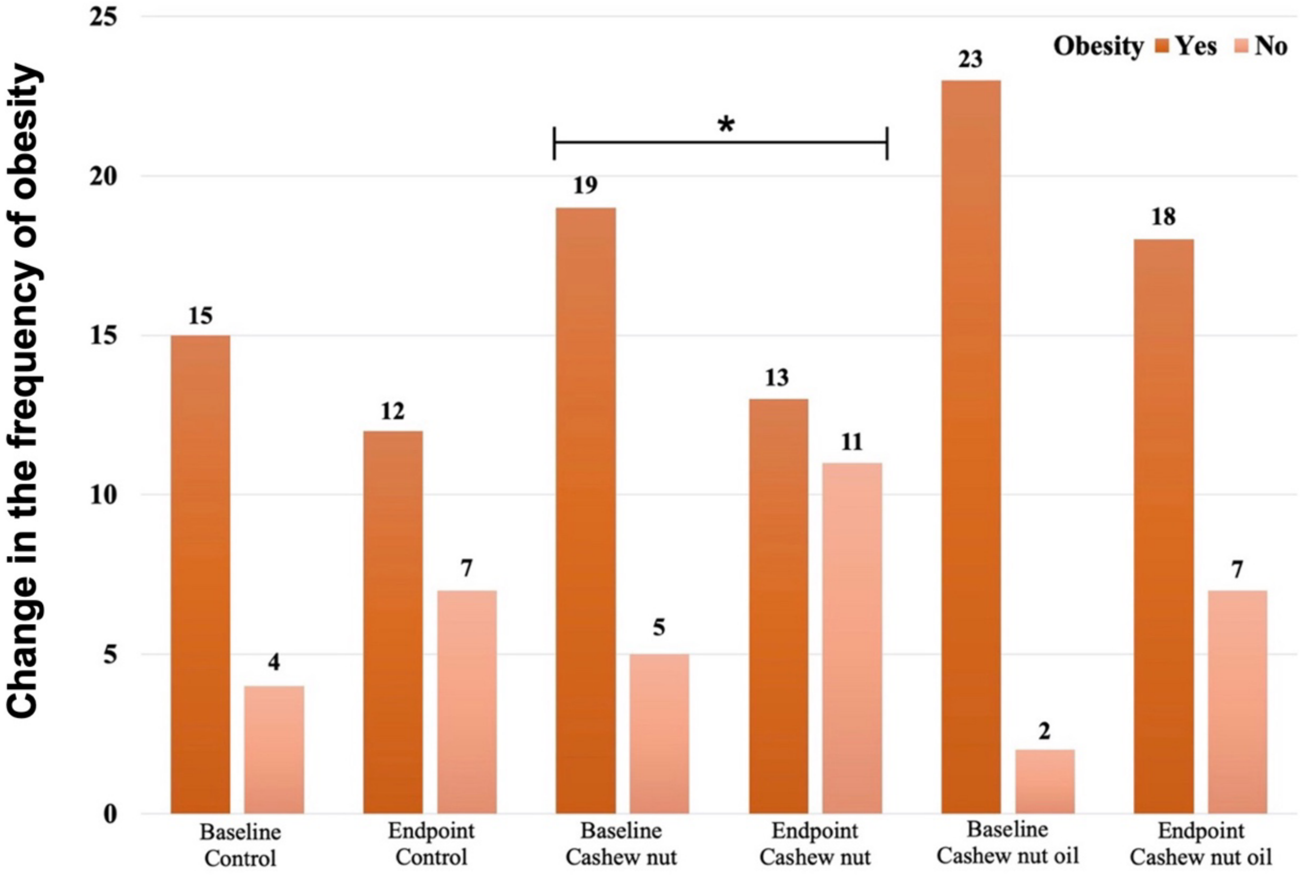

Supplementary Figure 3. Individuals with obesity according 8-wk energy-restricted groups (cashew nut, control, cashew nut oil). McNemar's test ($p < 0.05$ within-group).
